# Ribosomal protein S3: a critical regulator of human disease mechanisms

**DOI:** 10.1042/BSR20260051

**Published:** 2026-09-02

**Authors:** Joeline Dreik, Marwa Al Ali, Jalil Daher, Zeina Nasr

**Affiliations:** Department of Biology, Faculty of Arts and Sciences, University of Balamand, Tripoli, Lebanon

**Keywords:** cancer, DNA damage response, inflammation, NF-κB signaling, ribosomal stress, RPS3

## Abstract

Ribosomal protein S3 (RPS3) is an essential structural component of the 40S ribosomal subunit, yet growing evidence highlights crucial extraribosomal roles in genome maintenance, cell-cycle control, and immune signaling. Dysregulation of RPS3 contributes to diverse human disorders, including cancer, inflammatory diseases, neurodegeneration, and resistance to antimicrobial and anticancer therapies. As a cofactor of NF-κB and a participant in DNA damage responses, RPS3 occupies a node that integrates stress signaling with transcriptional reprogramming, enabling both protective and pathological outcomes. The present review critically evaluates mechanistic insights into RPS3 biology, emphasizing recent findings that delineate its context-dependent effects, discrepancies across models, and remaining gaps that restrict translational applications. Understanding these complexities is essential to assess RPS3′s potential as a biomarker and therapeutic target.

## Introduction

Ribosomal proteins are fundamental for ribosome biogenesis and translational accuracy, and mutations affecting their expression lead to disrupted hematopoiesis and increased cancer risk [1]. Ribosomopathies such as Diamond–Blackfan anemia and 5q-associated myelodysplastic syndrome illustrate how perturbations in ribosomal protein genes elicit tissue-specific disease despite global translational functions. The classical explanation involves activation of stress pathways—particularly the RPL5/RPL11-dependent p53 checkpoint—which suppresses cellular proliferation [2].

Among ribosomal proteins, ribosomal protein S3 (RPS3) stands out due to its multifunctional nature, encompassing both canonical ribosomal functions and diverse extraribosomal activities. Within the 40S ribosomal subunit, RPS3 is positioned at the beak region of the head of the small ribosomal subunit [3], where it contributes to translation initiation and quality control [4,5]. During 40S ribosomal subunit maturation, RPS3 undergoes a highly regulated assembly process involving sequential interactions with assembly factors. Following nuclear import, RPS3 initially associates with the chaperone Yar1, which maintains its N-terminal domain in rotated conformation. Yar1 is subsequently replaced by Ltv1, which stabilizes this intermediate state until phosphorylation-dependent release of Ltv1 enables the final incorporation of RPS3 into the 40S subunit and facilitates its interactions with rRNA and RPS20 required for stable ribosome formation [6]. Despite its essential role within the ribosome, a distinct non-ribosomal pool of RPS3 exists and can be functionally redirected in response to cellular stress. In particular, PKCδ-dependent phosphorylation of non-ribosomal RPS3 promotes its nuclear mobilization and enhances its DNA repair endonuclease activity, providing a mechanism through which RPS3 transitions between its roles in translation and genome maintenance [7]. Additionally, RPS3 regulates NF-κB-dependent gene expression, further highlighting its functional versatility beyond protein synthesis [8].

Recent studies further reveal diverse post-translational modifications (PTMs) of RPS3, including phosphorylation, arginine methylation, fucosylation, sumoylation, and ubiquitination, that dynamically regulate RPS3 stability, nucleolar import for 40S assembly, DNA repair endonuclease activity, and its transition between protein synthesis and stress-response functions [9–13]. Such versatility positions RPS3 as a key molecular mediator linking cellular stress to pathogenic signaling.

## RPS3 in DNA damage response, repair, and apoptosis

The earliest extraribosomal role of RPS3 is its involvement in the DNA damage response, where it functions as a context-sensitive regulator rather than a constitutive repair factor. Upon genotoxic stress, PKCδ phosphorylates RPS3, leading to its rapid translocation from the cytoplasm to the nucleus, a process that switches RPS3 from translational control and redirects it toward DNA damage-associated functions [14]. In the nucleus, RPS3 participates in nucleotide excision repair by interacting with the XPD helicase, thereby enhancing lesion recognition and unwinding at UV-induced cyclobutane pyrimidine dimers. These observations support a protective role for RPS3 under acute, localized DNA damage, particularly in proliferative cells where rapid repair is essential for genome stability [15].

In addition, RPS3 can repair mitochondrial DNA (mtDNA) damage. Under normal conditions, RPS3 is bound to Hsp90. But when the cells experience oxidative stress that induces mtDNA damage, RPS3 binds to heat shock protein Hsp70 instead. This binding, at the C terminus of the protein, exposes the mitochondrial targeting sequence (MTS) and helps with the translocation of RPS3 into the mitochondria. Once it reaches the outer membrane of the mitochondria, the MTS is recognized by the TOM70 protein of the translocator of the outer mitochondrial membrane (TOM) complex. This recognition enables RPS3 to reach the inner membrane, where it reduces the level of 8-oxo-guanine (8-oxo-G) in the damaged mtDNA due to its DNA damage repair endonuclease activity [16]. Mammalian RPS3 exhibits broad-spectrum DNA repair endonuclease activity, enabling it to recognize and cleave multiple forms of DNA damage, including thymine glycol lesions, apurinic/apyrimidinic sites, and UV-induced DNA lesions. Although RPS3 lacks DNA glycosylase activity, it repairs damaged DNA by cleaving the phosphodiester backbone adjacent to the lesion. In addition, RPS3 cleaves supercoiled UV-damaged DNA more efficiently than relaxed DNA [17].

Thus, subcellular localization of the RPS3 dictates its function beyond the ribosomes. Under oxidative stress, RPS3 translocation to the mitochondria contributes to the removal of 8-oxo-G lesions from mtDNA, thereby preserving mitochondrial genome integrity and bioenergetic function [18]. This mitochondrial role highlights a protective axis of RPS3 activity in post-mitotic or metabolically active cells, where mtDNA maintenance is critical for survival [19].

In other settings, RPS3 has an opposing role in DNA repair, where its interaction with the helicase RECQL4 inhibits helicase activity, delaying repair progression and potentially prolonging DNA damage signaling. This functional antagonism indicates that the outcome of RPS3 activity is determined by its binding partners and molecular context. This implies that RPS3 may act as a modulator that fine-tunes repair kinetics rather than as a unidirectional enhancer of repair. Such inhibition may be adaptive under conditions where damage exceeds repair capacity, allowing checkpoint activation or cell fate decisions to proceed [20]. This underscores the need to define the determinants that shift RPS3 between protective and detrimental activities.

The same context dependence applies to the effect of RPS3 on apoptosis. RPS3 contains two distinct functional domains: a C-terminal domain responsible for DNA repair and an N-terminal domain that mediates apoptosis. Overexpression of RPS3 induces apoptosis through its translocation from the cytoplasm to the nucleus and activation of the caspase-8/caspase-3 signaling pathway, with RPS3 acting upstream of both caspases. In addition, this translocation is enhanced by cytokine stimulation, particularly by TNF-α as opposed to Fas ligand, suggesting that RPS3 sensitizes cells to cytokine-mediated apoptosis [21]. Similarly, RPS3 can induce programmed cell death through activation of the JNK pathway and the association with TRADD, promoting caspase-dependent apoptosis under conditions of irreparable damage [22]. In contrast, under basal or low-stress conditions, RPS3 is actively restrained through HSP70-CHIP-mediated ubiquitination and proteasomal degradation, preventing inappropriate activation of apoptotic signaling [23]. These opposing effects of RPS3 as a threshold-dependent switch enable cell survival when damage is limited while activating apoptosis when damage is severe.

Collectively, these findings support a model in which RPS3 can be considered as a critical regulator for DNA damage response and apoptosis. The effect of RPS3 depends on the cell type, severity and duration of stress, subcellular compartmentalization, and PTMs. For instance, under mild or transient DNA damage, RPS3 promotes repair through its function as an endonuclease and its ability to interact with proteins involved in excision repair. While under severe damage, RPS3 can induce cytochrome c (Cyt c) release and activate caspases, leading to apoptosis. Dysregulation of these determinants may shift RPS3 from a genome-protective factor to a pathogenic contributor, with implications for cancer, neurodegeneration, and aging.

## RPS3 in host defense and immune signaling

RPS3 has been linked to a variety of immunological responses through its role as a regulator of NF-κB and its involvement in host response to bacterial and viral infections. Its extraribosomal role makes it a potential biomarker for multiple inflammatory disorders and a selective regulator of specific immune responses, which are tissue, cell, and context-dependent. This property underlies its dual capacity to enhance host defense or drive immunopathology.

At the molecular level, RPS3 is considered a non-Rel component of the NF-κB complex that is composed of p50 and p65 (RelA) subunits. RPS3 interacts with the p65 subunit to increase the affinity of NF-κB for a restricted subset of κB promoters, thereby amplifying the expression of pro-inflammatory genes [8]. In immune cells such as macrophages and epithelial cells, transient nuclear accumulation of RPS3 following pathogen sensing enhances antimicrobial gene expression and supports effective early host defense.

**Figure 1:**
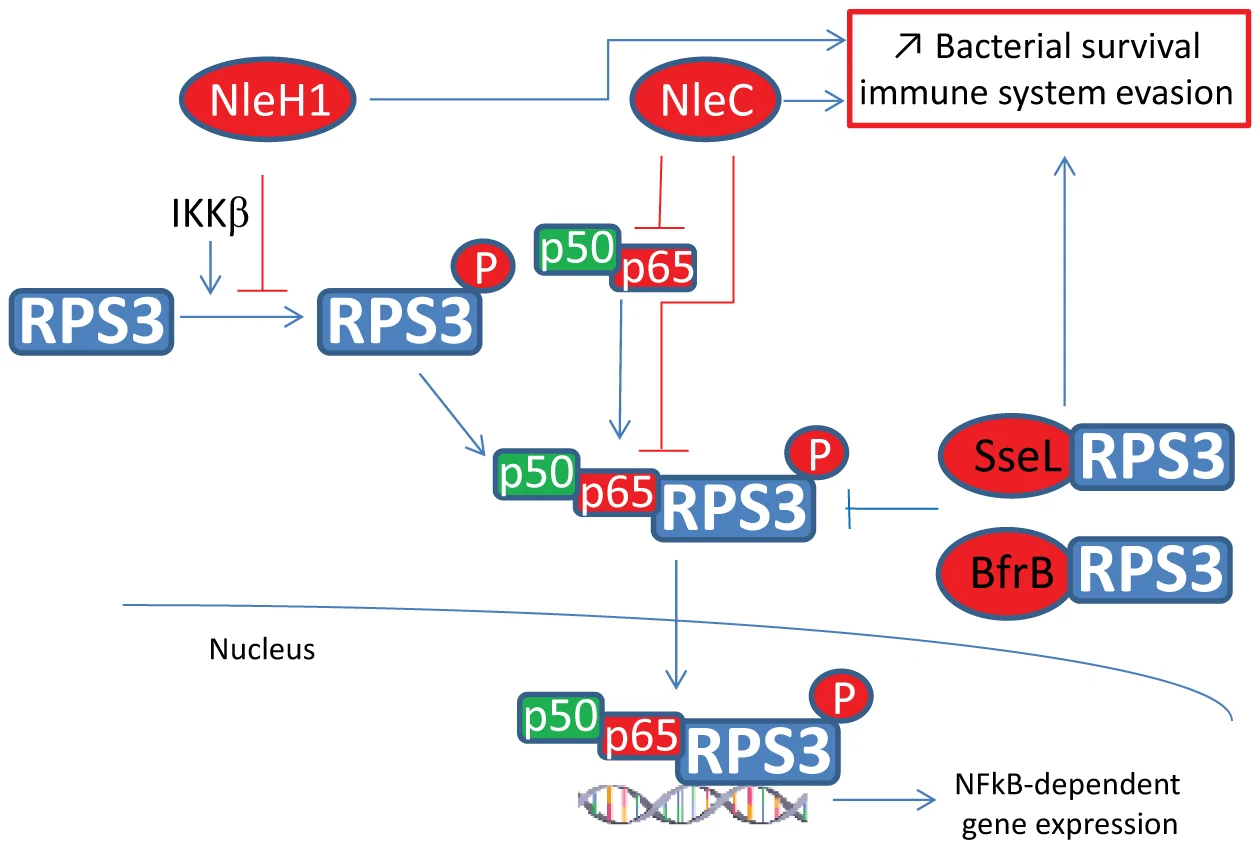
The role of RPS3 in immune regulation The major pathways through which RPS3 modulates the immune system and helps in bacterial survival within the host cells. Among these, the effector protein NleH1 inhibits IKKβ-mediated phosphorylation of RPS3, while NleC cleaves p65 and inhibits the formation of the RPS3-p50-p65 complex, leading to suppression of NF-κB signaling. Other bacterial effectors, including SseL and BfrB, directly interact with RPS3, impairing host defense mechanisms. These interactions inhibit RPS3-dependent NF-κB activation, which down-regulates antimicrobial and pro-inflammatory responses. These interactions contribute to immune system evasion and bacterial survival within host cells.

However, this same mechanism is tightly regulated and frequently exploited by pathogens, highlighting the importance of subcellular localization and protein-protein interactions as determinants of RPS3 function. Multiple bacterial effectors, such as NleH1, NleC, secreted effector L (SseL), and BfrB, pecifically prevent RPS3 nuclear translocation or disrupt RPS3-p65 complexes. For instance, following NF-κB stimulation, IKKβ binds to RPS3 and phosphorylates it, mediating its translocation into the nucleus. This phosphorylation has been shown to be specifically inhibited by the *Escherichia coli* O157:H7 effector NleH1 both *in vitro* and *in vivo*. This is followed by the suppression of RPS3-dependent NF-κB target gene expression, including IL-8 and TNF-α, without affecting p65 nuclear translocation, thereby enhancing bacterial colonization and diarrhea while limiting host mortality [24]. In addition, the metalloprotease NleC, secreted by attaching/effacing pathogens, suppresses host NF-κB-driven inflammatory responses by targeting and cleaving a small part of p65. This fragment specifically binds to RPS3, thereby preventing RPS3 from translocating into the nucleus. The disruption of the RPS3-dependent NF-κB signaling pathways significantly weakens proinflammatory gene expression and immune cell infiltration, allowing bacterial pathogens to evade host immune defenses [25]. Similarly, both *Salmonella enterica* and *Mycobacterium tuberculosis* employ the SseL [26] and the ferritin-like protein BfrB [27], respectively, to directly bind RPS3, preventing its nuclear translocation after TNF-α activation. This inhibition impedes TNF-α-induced NF-κB-dependent genes such as IL-2 and IL-8, which are involved in the host’s immune response to infection (Figure 1).

Notably, these strategies dampen host inflammatory responses without completely abolishing NF-κB signaling. This suggests that pathogens exploit RPS3's role as a signal amplifier to evade immunity while minimizing host lethality. In this context, limiting RPS3 activity may paradoxically preserve tissue integrity and host survival [25–28] (Figure 1). Conversely, RPS3 can actively promote inflammatory cell death under certain conditions. During *Yersinia* infection, glutathionylated LcrV binds RPS3 and redirects its function toward inflammasome-associated macrophage pyroptosis, resulting in exaggerated cytokine release and tissue damage [29]. This shift clearly indicates that RPS3 can function as a transcriptional co-regulator or a mediator of inflammatory injury depending on the stress intensity and the pathogen-derived PTMs of interacting proteins.

Disease-specific studies further highlight the importance of cell type and inflammatory environment in determining the effects of RPS3. In chronic airway diseases such as allergic asthma and COPD-like inflammation, sustained overexpression of RPS3 in airway epithelial and immune cells activates NF-κB signaling, contributing to persistent inflammation and tissue remodeling [30,31]. In contrast, during the resolution phase of inflammatory arthritis, overexpression of RPS3 in macrophages induces IL-10 production and therefore anti-inflammatory signaling, facilitating tissue repair and immune homeostasis [32]. These opposing effects demonstrate that expression levels as well as cell context determine the functional outcome of the protein.

In addition to intracellular signaling, RPS3 can also exert extracellular immune-modulatory functions. Its release from dying cancer cells enables activation of TLR4 on dendritic cells, enhancing antigen presentation and antitumor immunity. This noncanonical role underscores how cell death modality and extracellular localization can convert RPS3 into a danger-associated molecular signal with promising therapeutic implications [33].

Collectively, these findings support a model in which RPS3 serves as a context-dependent immune regulator, integrating pathogen signals, stress intensity, post-translational regulation, and cell-type-specific signaling networks to determine immune outcomes. While transient, spatially restricted RPS3 activation promotes effective host defense, sustained or ectopic activity can drive chronic inflammation or inflammatory cell death.

Collectively, the data presented supports the hypothesis that RPS3 functions as an immune checkpoint by inducing NF-κB signaling in a context-dependent manner. Its effects depend on its subcellular localization, interacting proteins, and duration of signaling. Under acute infection, this mechanism promotes host defense, while chronic infection can induce inflammation. This model predicts that the activity of RPS3 can be selectively modulated in specific immune cell settings, which may provide greater therapeutic benefit than general inhibition of NF-κB signaling.

## RPS3 in cancer development and progression

RPS3 is known to be secreted as a homodimer into the extracellular environment by cancer cells. Its secretion is markedly increased in highly malignant and drug-resistant cancer cells compared with less aggressive and normal cells, indicating a potential association between extracellular RPS3 levels and cancer malignancy [34]. Interestingly, RPS3 is known to undergo N-linked glycosylation at its Asn165 residue, which allows its secretion via the ER-Golgi pathway in various cancer cell lines. The secreted RPS3 can homo-dimerize, and mutating its Asn165 residue reduces its secretion, which correlates with a reduction in cell migration and invasion *in vitro*. Its elevated secretion in highly invasive cancer cells suggests its potential involvement in tumor progression and malignancy [35].

**Figure 2:**
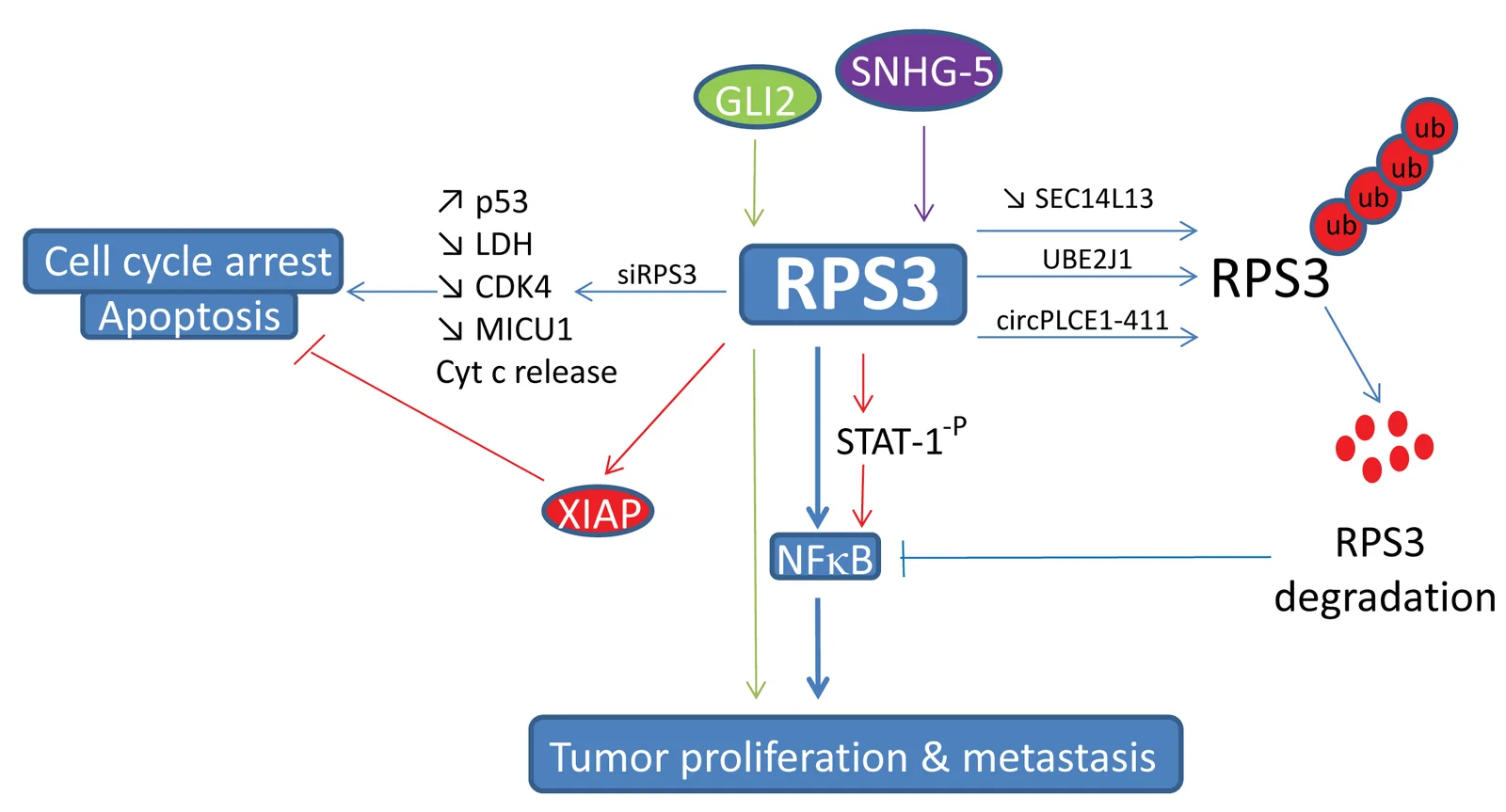
The role of RPS3 in tumorigenesis The key oncogenic pathways in which RPS3 plays a role, include the effects of the ribosomal protein on cell proliferation, cell cycle progression, apoptosis, and metastasis. In fact, overexpression of RPS3 has been linked with increased tumor proliferation and metastatic potential in multiple cancer types. Through its interaction and activation of the NF-κB pathway, RPS3 induces the transcription of genes involved in oncogenic signaling. RPS3 stability is regulated by several upstream factors, including SEC14L3, UBE2J1, and circPLCE1-411, which modulate RPS3 ubiquitination and proteasomal degradation. Inhibition of these regulatory mechanisms results in RPS3 accumulation and enhanced oncogenic activity. On the other hand, down-regulation of RPS3 through siRNA suppresses tumor-promoting pathways and induces anticancer effects through multiple mechanisms; RPS3 depletion increases p53 activity, leading to cell cycle arrest and growth inhibition. Its knockdown reduces the expression of lactate dehydrogenase (LDH), cyclin-dependent kinase 4 (CDK4), Cyt c, and mitochondrial calcium uptake protein 1 (MICU1), which impair cellular metabolism, cell cycle progression, and mitochondrial function. RPS3 can also modulate various oncogenic factors such as XIAP and STAT1 and is itself regulated by Gli-2 and SNHG5, which contribute to enhanced cell survival, resistance to apoptosis, tumor growth, and metastatic behavior.

In fact, most cancers display elevated RPS3 expression associated with poor clinical outcomes. However, accumulating evidence indicates that RPS3 does not function as a uniform oncogenic driver. Instead, its tumor-associated effects reflect context-dependent integration of survival signaling, DNA damage responses, and apoptotic thresholds, with outcomes governed by cell types, oncogenic background, and stress exposure. Mechanistically, RPS3 promotes tumor behavior through the different pathways listed below (Figure 2).

### Pro-growth and survival signaling

In many cancer types, RPS3 stabilizes NF-κB transcriptional activity, thereby enhancing pro-survival gene expression. This activity is particularly evident in cancers characterized by chronic inflammatory signaling or high basal stress, where RPS3 nuclear localization amplifies expression of anti-apoptotic and metabolic genes. In colorectal cancer (CRC), RPS3 knockdown suppresses growth through p53 induction and metabolic rewiring through LDH suppression, indicating that RPS3 contributes to tumor cell survival by buffering genotoxic and metabolic stress [36]. Pharmacological inhibition by salicylic acid can mimic RPS3 knockdown, leading to the same outcome: G1-phase cell cycle arrest and reduced CDK4 expression, a key regulator of the G1-to-S phase transition. These data support a functional dependency on RPS3 in this context [37]. Also in CRC, targeting RPS3 for degradation by ubiquitination has been demonstrated to induce its dissociation from NF-κB, leading to the reduction of the nuclear translocation of the latter and a decrease in tumor progression. The ubiquitin-dependent degradation of RPS3 has been shown to be promoted by either the ubiquitin-conjugating enzyme UBE2J1 [38] or the circular RNA (circRNA)-encoded protein circPLCE1-411 [39]. Similarly, in clear cell renal cell carcinoma tissues, suppression of SEC 14L3 can induce RPS3 ubiquitination and degradation, leading to the same effects [40].

In addition, in melanoma, knockdown of RPS3 suppresses cell growth and colony formation *in vitro* while reducing tumor growth *in vivo*. Mechanistically, Tian et al. have shown that RPS3 controls melanoma cell survival through the mitochondrial signaling pathway. RPS3 knockdown induces the opening of the mitochondrial permeability transition pore, resulting in excessive calcium influx into the mitochondria and significant cytochrome c efflux into the cytoplasm, inducing the activation of caspases. This correlates with a down-regulation of the expression of MICU1, an important gatekeeper for calcium uptake into the mitochondria. These changes ultimately impede melanoma cell growth and induce apoptosis [41].

Importantly, this pro-survival role of RPS3 likely reflects stress intensity and repair capacity. In cancer cells exposed to DNA damage or genotoxic stress, RPS3 may have a role in inducing proliferation by shifting from facilitating genome maintenance to enabling damage tolerance. This indicates that RPS3′s DNA repair functions that are protective in normal cells may become pathogenic in tumors by sustaining survival beyond tolerable damage thresholds.

### Metastasis and invasion

Accumulating evidence implicates RPS3 as a driver of tumor progression, invasion, and metastasis through both metabolic and transcriptional reprogramming. In hepatocellular carcinoma (HCC), RPS3 interacts with the chaperonin CCT6A—an adverse prognostic factor—to promote metastatic behavior, potentially by rewiring lipid, amino acid, and serine metabolism, as CCT6A silencing markedly reduces HCC cell migration, invasion, and proliferation [42]. Complementarily, oncogenic activation of RPS3 in HCC can also occur via the lncRNA SNHG5, which enhances RPS3 expression and stimulates NF-κB signaling, thereby promoting tumor growth and resistance to apoptosis both *in vitro* and *in vivo* (Figure 2) [43]. Beyond HCC, elevated RPS3 expression in adenoid cystic carcinoma correlates with poor prognosis and metastatic dissemination, where RPS3 drives migration, invasion, and cisplatin resistance through STAT1 phosphorylation and NF-κB activation; notably, RPS3 knockdown suppresses tumor growth and metastasis *in vivo* (Figure 2) [44]. In addition, RPS3 has been identified as a downstream target gene for GLI2 in osteosarcoma. GLI2 is a transcription factor known to be associated with the proliferation and invasion of osteosarcoma and has been correlated with poor prognosis. RPS3 is up-regulated in highly invasive osteosarcoma cells compared with non-invasive ones, with its levels further increasing in lung metastases. As a downstream target gene of the transcription factor GLI2, its expression is up-regulated following GLI2 activation and down-regulated after its inhibition. By promoting invasion and metastasis, RPS3 plays a key role in osteosarcoma progression [45]. Collectively, these findings position RPS3 as a multifunctional oncogenic regulator linking metabolic adaptation and pro-invasive signaling to cancer progression.

### Regulatory complexity and contradictions

Adding further complexity, RPS3 also exerts post-transcriptional and NF-κB-independent functions. In breast cancer, RPS3 stabilizes XIAP independently of inflammatory signaling, directly suppressing apoptosis [46], suggesting that targeting RPS3 may influence apoptosis independent of inflammation. Such findings indicate that RPS3 can bypass transcriptional control to regulate cell fate at the protein stability level. These alternative mechanisms point to how post-translational regulation and protein-protein interaction determine whether RPS3 activates survival or permits apoptosis.

Altogether, these interpretations propose that RPS3 is not universally oncogenic but can emerge as a context-dependent oncogenic vulnerability. Its pathogenicity largely depends on the balance between survival and apoptotic signals, which varies widely across tumor types. We hypothesize that RPS3 represents a context-dependent pan-cancer regulator through its extraribosomal functions with its ability to induce survival while modulating apoptosis.

### RPS3 as pan-cancer regulator

The effect of RPS3 in a wide range of cancer types, including colorectal, hepatocellular, melanoma, osteosarcoma, adenoid cystic carcinoma, breast, and renal cell carcinoma, proposes that its involvement in tumor onset and development is not restricted to a single tissue type. Although these tumors are highly heterogeneous, common roles of RPS3 include NF-κB signaling activation, cell proliferation and survival induction, DNA damage response regulation, apoptosis modulation, and metastasis induction. All these data suggest that RPS3 may be a pan-cancer regulator through its various extraribosomal functions, including the modulation of stress responses, DNA repair pathways, inflammatory signaling, and apoptotic regulation. Importantly, the role of RPS3 seems to be tissue and context-specific depending on the specific downstream effectors involved. Whether RPS3 functions as a general effector across various cancers or as a specific modulator for oncogenic subgroups needs further elucidation through transcriptomic, proteomic, and functional studies in the future.

## RPS3 in chemotherapy and radioresistance

The involvement of RPS3 in therapeutic resistance further represents the dual nature of the protein. Accumulating data show that RPS3 promotes resistance to multiple therapies. In many cancers, RPS3 promotes resistance by enhancing stress-adaptive signaling following treatment. It enhances NF-κB transcription following radiation, enabling NSCLC cell survival [47]. In addition, Lyn-mediated phosphorylation increases MDR1 translation, linking RPS3 to multidrug resistance [48]. While loss of negative regulators (e.g., SIAH1 in ovarian cancer; TRIP13 facilitation in myeloma) sustains RPS3 and therapeutic escape [49,50], exosome transfer of RPS3 induces cisplatin resistance between gastric cancer cells [51]. In these settings, RPS3 seems to switch the therapy-dependent stress into a survival signal by inducing DNA damage tolerance and inhibiting apoptosis. This supports its oncogenic role in stress-induced settings and suggests that under conditions of acute sublethal stress, RPS3 shifts damage responses toward survival rather than apoptosis.

Paradoxically, the opposite scenario is observed in glioblastoma, where RPS3 degradation by RNF138—rather than its stabilization—enhances resistance by suppressing apoptotic signaling [52]. This suggests that RPS3 contributes to therapy response in a pathway-selective manner. The major challenge remains whether RPS3 expression can predict drug response in clinical cohorts, and this question is still largely untested and needs further elucidation in the future.

## RPS3 in neurological and other disorders

RPS3 is broadly expressed across multiple brain regions, where its extraribosomal functions appear to be tightly linked to neuronal DNA damage responses and apoptotic control. Unlike proliferative tissues, neurons rely heavily on efficient repair and survival mechanisms, making the regulation of RPS3 expression particularly significant in the nervous system.

In the hippocampus, RPS3 expression declines with age and chronic stress, a pattern that associates with decreased capacity for DNA repair and impaired neuronal elasticity [53]. Since it is already established that RPS3 plays a role in repair of oxidative and genotoxic lesions, this age-associated reduction likely compromises genome maintenance in post-mitotic neurons, thereby increasing vulnerability to cumulative damage. In this context, it appears that RPS3 performs a protective function, supporting neuronal survival under moderate stress conditions.

However, RPS3 can also induce neuronal apoptosis depending on stress intensity and subcellular localization. In cultured neurons exposed to acute or severe stress and in ischemic hippocampal neurons, overexpressing RPS3 has been shown to activate pro-apoptotic signaling pathways and induce neuronal cell death [54–56], consistent with its ability to induce JNK- and caspase-dependent cell death in other systems. These findings suggest that upon increased stress intensity and when damage exceeds repair capacity, RPS3 may participate in threshold-dependent elimination of irreversibly damaged neurons, a process that could be protective at the tissue level but detrimental when excessive.

These contradicting effects are further illustrated in Parkinson's disease models, where exogenous therapeutic delivery of RPS3 protects dopaminergic neurons from degeneration. Notably, this protective role is limited to specific neuron subtypes and disease stages [57]. These findings suggest that the dual role of RPS3 highly depends on the cell type, signaling networks, and the temporal progression of stress as shown in other settings. Early or moderate mitochondrial and oxidative stress may favor mitochondrial or nuclear roles of RPS3 in DNA repair and stress adaptation, whereas late-stage or overwhelming stress may redirect RPS3 toward apoptotic pathways.

In addition, recent transcriptomic studies identify RPS3 as a potential diagnostic biomarker for acute ischemic stroke, a condition characterized by abrupt, high-intensity oxidative and genotoxic stress [58,59]. Although functional data *in vivo* are limited, it is plausible that RPS3 participates in early damage responses that attempt to preserve neuronal integrity, while prolonged ischemia or reperfusion injury could shift its role toward apoptotic signaling. This highlights the importance of temporal dynamics and post-translational regulation—such as phosphorylation or proteasomal turnover—in dictating RPS3 outcomes during acute neurological injury.

Beyond the nervous system, associations between RPS3 and metabolic or cardiovascular disorders have been reported, though findings remain inconsistent. In one study, RPS3 has been demonstrated as a hub gene in coronary artery disease (CAD) while investigating the role of circRNAs in the progression of CAD through bioinformatics analysis [60]. Similarly, RPS3 has been found overexpressed in a proteomic analysis of mice with type 2 diabetes compared with normal control [61]. Conversely, another study has found that the levels of RPS3 are significantly reduced in diabetic obese individuals compared with their non-diabetic counterparts [62]. These preliminary findings show a link between RPS3 and diabetes, but more research is needed to decipher the role of RPS3 in this particular context. These discrepancies may reflect differences in tissue-specific expression, systemic stress levels, and inflammatory burden, as well as uncharacterized PTMs that influence RPS3 stability and localization. Unlike cancer or immune cells, metabolic tissues may engage RPS3 primarily in stress adaptation and survival rather than proliferation, further underscoring the importance of physiological context.

Collectively, available evidence suggests that RPS3 acts as a context-dependent regulator of cellular fate in neurological and systemic disorders, integrating DNA repair capacity, apoptotic thresholds, and stress signaling. A major unresolved challenge is to define how these regulatory layers operate *in vivo*, particularly in chronic and age-related diseases, where subtle dysregulation of RPS3 may have cumulative pathological consequences.

## Conclusion

RPS3 integrates core aspects of translational control with DNA repair, apoptosis, immune activation, and cellular metabolism. Recent work underscores its prominent role as a disease modifier in cancer, infection, and neuroinflammation. However, contradictory findings—particularly regarding therapy resistance and DNA repair efficiency—highlight complex regulatory networks that remain insufficiently defined.

Future translational success requires mapping context-specific interactomes of RPS3 in disease stages and understanding tissue-specific functions and post-translational regulation. Additionally, it may require evaluating RPS3 as a biomarker in prospective clinical studies, especially when developing selective modulators that could help in distinguishing protective versus pathogenic RPS3 activities. Overall, RPS3 represents a compelling yet under-validated therapeutic target. A deeper mechanistic understanding will clarify whether intervention should aim to inhibit, stabilize, or redirect its functions depending on rthe pathological setting.

## Abbreviations

8-oxo-G: 8-oxo-guanine
CAD: coronary artery disease
CDK4: cyclin-dependent kinase 4
circRNA: circular RNA
CRC: colorectal cancer
Cyt c: cytochrome c
HCC: hepatocellular carcinoma
LDH: lactate dehydrogenase
MICU1: mitochondrial calcium uptake protein 1
mtDNA: mitochondrial DNA
MTS: mitochondrial targeting sequence
PTMs: post-translational modifications
RPS3: ribosomal protein S3
SseL: secreted effector L
TOM: translocator of the outer mitochondrial membrane
